# Lengthening Patients Previously Treated for Massive Lower Limb Reconstruction for Bone Tumors with the PRECICE 2 Nail

**DOI:** 10.3390/children10111772

**Published:** 2023-10-31

**Authors:** Laura Campanacci, Luca Cevolani, Marco Focaccia, Giovanni Luigi Di Gennaro, Barbara Dozza, Eric Staals, Federica Zuccheri, Giuseppe Bianchi, Davide Maria Donati, Marco Manfrini

**Affiliations:** 1 3rd Orthopaedic and Traumatologic Clinic Prevalently Oncologic, IRCCS Istituto Ortopedico Rizzoli, Via Pupilli 1, 40136 Bologna, Italy; laura.campanacci@ior.it (L.C.); marco.focaccia@ior.it (M.F.); eric.staals@ior.it (E.S.); federica.zuccheri@ior.it (F.Z.); giuseppe.bianchi@ior.it (G.B.); davidemaria.donati@ior.it (D.M.D.); marco.manfrini@ior.it (M.M.); 2 Department of Pediatric Orthopedics and Traumatology, IRCCS Istituto Ortopedico Rizzoli, Via Pupilli 1, 40136 Bologna, Italy; giovanniluigi.digennaro@ior.it; 3 Department of Biomedical and Neuromotor Sciences (DIBINEM), Alma Mater Studiorum University of Bologna, 40126 Bologna, Italy; barbara.dozza@ior.it

**Keywords:** bone tumor, PRECICE 2 nail, bone tumor, osteosarcoma, limb length discrepancy, magnetic nail

## Abstract

The objective of this study was to determine the efficacy of the PRECICE 2^®^ nail in the treatment of lower limb length discrepancy in patients with a history of bone tumors. This study reports on outcomes, complications, and the safety of the PRECICE 2 limb lengthening nail in a cohort of pediatric patients with limb length discrepancy after surgery for bone tumors. Seventeen patients were treated with intramedullary magnetic nails. The average patient age at the time of surgery was 19 (range 11–32). The PRECICE 2 nail was used on 14 femurs (6 retrograde and 8 anterograde) and 3 tibias. The average consolidation time was 141 days (range 50–360) with a mean CI of 31 ± 12 days/cm. The ASAMI bone score showed 14 (82%) excellent results, 1 (6%) good result, and 2 (12%) poor results. The ASAMI functional score showed 13 (84.6%) excellent results, 3 (11.5%) good results, and 1 (3.8%) fair result. Patients treated with chemotherapy for bone cancer did not show any increase in distraction time or consolidation time. A total of 3 (17%) problems, 1 obstacle (5.5%), and 1 complication (5.5%) were encountered in our case series. The PRECICE 2 nail allows for effective and accurate lengthening preserving the range of motion in patients treated for bone tumors.

## 1. Introduction

Skeletal reconstruction after resection of bone tumors in the lower extremities of children remains a difficult challenge. Most primary bone tumors arise in children and adolescents and are located around the knee (proximal tibia and distal femur). The distal femoral physis accounts for approximately 70% of the overall growth of the femur (40% of the total growth of the entire lower extremity), while the proximal physis of the tibia contributes to approximately 55% of the total growth of the tibia (25% of the overall growth of the lower extremities) [1]. Limb salvage can be achieved in most cases, with neoadjuvant chemotherapy. The type of reconstruction depends on the site and extent of the tumor, and in children, it strictly depends on the relationship of the tumor with the growth plate. If the tumor extends to the metaphysis and/or to the epiphysis, resection may sacrifice the growth plate of the affected bone, and articular reconstruction with a standard adult-type prosthesis sterilizes the growth plate of the opposite bone segment as well, thus causing a discrepancy that will be greater the younger the child is at the time of surgery [2,3].

In the era of limb salvage, the gold standard to treat bone tumors in children should preserve not only the limb itself but also its function. Equal limb length at maturity and good functional outcome are the main goals of these surgeries but are difficult to achieve. Various techniques have been proposed to limit the final length discrepancy and to preserve the patient’s bone stock, such as osteoarticular allografts, hemi-articular composite prostheses [4], and expandable prostheses [5]. Biological reconstructions may preserve and replace the bone stock, but they leave the issue of longitudinal growth unresolved. Expandable prostheses may require repeated surgeries, induce gradual bone stock loss in the affected limb, and require a minimum amount of space to insert the prosthesis and the stems.

During skeletal growth, one option to minimize length discrepancy is to reduce the growth of the contralateral limb with epiphysiodesis, which is limited by the patient’s growth potential and therefore often does not guarantee optimal correction of the length difference.

Once skeletal growth has stopped, bone lengthening with callus distraction can correct limb length discrepancy (LLD). External fixation has been the gold standard for limb lengthening thus far. Nevertheless, the use of an Ilizarov frame or another external fixator device is plagued by several disadvantages, such as pin tract infection [6,7]; risk of fracture at the regeneration site after removal [8]; pain, stiffness, and muscle contracture due to soft tissue fixation by pins or Kirshner wires [9]; and the patient’s inconvenience of having to accept long-term, bulky, and disabling devices [10].

Intramedullary motorized lengthening devices (powered by magnetic or electrical energy as opposed to devices requiring mechanical action) have generated new possibilities for reconstruction. Moreover, they allow for earlier rehabilitation, have a lower infection risk, and are received better by patients [11,12,13,14,15] when compared to external fixators.

The use of a magnetic extendable nail is reported in the literature as safe and effective. However, there are few papers that describe its use in patients with a history of bone tumors. The PRECICE 2 nail (NuVasive, Inc., San Diego, CA, USA) is a magnetically driven telescopic titanium nail that lengthens or shortens with an internal magnetic gearbox activated by an external magnetic field [16].

Usually, in patients with LLD, bone lengthening is performed through the native skeleton, while in patients previously treated for bone tumors, the discrepancy must be managed considering the previous skeletal reconstruction, which is usually accomplished using bone allografts and long synthesis devices, megaprostheses, or composite allo-prosthetic reconstruction.

Therefore, preoperative planning in this group of patients should first consider the previous reconstruction to determine the surgical approach, the nail size, and the site of osteotomy.

In this study, we present a series of patients with LLD secondary to surgical treatment for bone tumors during childhood, which is the largest described up to now. We, therefore, undertook a retrospective analysis of a consecutive cohort to determine the following: (1) whether limb length discrepancy of the lower limbs in patients treated for bone tumor during childhood may be safely performed using a magnetic intramedullary nail (PRECICE 2); (2) whether patients treated with chemotherapy for bone sarcoma experienced a risk profile similar to others; (3) whether the type of reconstruction for a bone tumor influences the lengthening strategy.

## 2. Materials and Methods

A retrospective study was performed to identify all consecutive cases of LLD secondary to surgery for a bone tumor treated with the PRECICE 2 nail at our Institute from January 2014 to January 2022.

Inclusion criteria were as follows: (1) patients treated for benign or malignant bone tumors in childhood; (2) LLD ≥ 30 mm; (3) follow-up ≥ 12 months after the end of lengthening.

Exclusion criteria were as follows: (1) disease progression; (2) LLD secondary to treatment for a benign or malignant bone tumor after skeletal growth had stopped.

The Local Ethics Committee approved this study (10/2014/Oss/IOR; 27 June 2014), and informed consent was obtained from all patients.

### 2.1. Planning

The first surgery influences the lengthening strategy. Each patient underwent a case-by-case evaluation, with the aim of preserving the previous reconstruction and obtaining the best lengthening. Based on the site of the previous treatment, we identified 3 scenarios:

1. Tumor involving proximal and distal femur, or distal tibia: lengthening was performed on the affected bone opposite to the previous surgery.
2. Tumor involving proximal tibia: lengthening was performed on the proximal femur.
3. Tumor involving the pelvis: lengthening was performed distally to the femur.

Two cases in our cohort were treated differently. One patient treated for proximal tibial tumor was lengthened distally to the tibia. The patient presented external sciatic popliteal nerve paralysis and ankle anchylosis after the first surgery. A retrograde straight femoral nail was applied distally to the tibia and through the calcaneal talus joint.

Another patient presented bone hypermetria after treatment for distal femur osteosarcoma with a rotationplasty. In this case, the contralateral femur ended up shorter than the treated bone. To restore knee alignment, lengthening was performed on the untreated femur.

### 2.2. Patients

The accurate distraction length and the appropriate size and diameter of the nail were determined case by case with a plain radiograph and physical examination. The surgical technique has been described previously by Paley [17]. All patients began active and passive mobilization of lower limb joints, and walking with toe-touch weight bearing was permitted the day after surgery. Lengthening started 5–7 days after surgery, and two lengthening schedules were performed as follows:

- 1 mm per day in 3 steps of 0.33 mm for femoral lengthening or, in cases of planned lengthening, <6 cm;
- 0.66 mm per day in 2 steps of 0.33 mm for tibial lengthening or, in cases of planned lengthening, >6 cm.

In cases of joint stiffness or delayed ossification at the regeneration site, lengthening was slowed down during treatment. After 3 days of lengthening, an X-ray was performed to confirm the correct functioning of the nail, and then patients were discharged.

Patients visited the outpatient clinic for wound review 10 days after beginning lengthening, and an X-ray was acquired to check that lengthening was correctly performed. A review with a plain radiograph occurred bi-weekly during lengthening, monthly during the consolidation phase, and every 3 months thereafter. Partial weight bearing (25% of body weight) was allowed during the consolidation phase.

### 2.3. Follow-Up

The outcomes measured were the Distraction Index (DI is the ratio between the number of days of distraction and the achieved length in cm) and the Consolidation Index (CI is the ratio between the number of days from surgery to consolidation and the achieved length in cm). The distraction time was calculated from surgery to the end of lengthening. The consolidation time was defined as the time it took time to obtain bone callus from surgery to callus formation along all four cortices of long bone and painless full weight bearing.

Three of the researchers (CLu, BG, SE) independently evaluated the X-ray. The presence of a bridge callus in 3 out of 4 cortices in antero-posterior and lateral X-ray images and painless weight bearing 6 months after the end of the distraction phase were considered parameters for bone healing. The senior researcher (CLa) confirmed all the evaluations. Delayed union was defined as no progression in new bone formation on two consecutive X-rays 1 month apart.

Postoperative complications were classified according to the Paley classification [9] as follows:

- Problem (postoperative difficulty that resolved completely without intervention);
- Obstacle (difficulty that required surgery yet resolved completely afterward);
- True complication (intra- or postoperative complication that remained unresolved even after treatment was completed).

Clinical results were classified according to the Association for the Study and Application of the Methods of Ilizarov (ASAMI) criteria. This score classifies bony and functional results as excellent, good, fair, and poor. The criteria for establishing the bony results were as follows:

- Union;
- Infection;
- Residual deformity;
- Limb length discrepancy.

The criteria for functional results were limping, stiffness of either the knee or the ankle, sympathetic dystrophy, pain that resulted in reduced activity or disturbed sleep, and inability to return to previous activities (ASAMI classification).

Descriptive statistics were created for all variables. A chi-squared test was used to test the association between categorical variables. For abnormally distributed data, the Mann–Whitney test was used to analyze two independent populations. Significance was set at *p* < 0.05 in all statistical analyses. All statistical analyses were performed with IBM SPSS Statistic 21.0 (IBM™ Corp, Armonk, NY, USA).

## 3. Results

A total of 41 patients were treated at our institution with the PRECICE 2 intramedullary nail. Eighteen of them fit the inclusion criteria. The underlying etiology of LLD is reported in Table 1.

One patient underwent lengthening twice. Three patients were previously treated with contralateral epiphysiodesis (proximally to the tibia and distally to the femur in two cases, and distally to the femur in one). Ten patients underwent chemotherapy previously.

### 3.1. Lengthening

**Figure 1:**
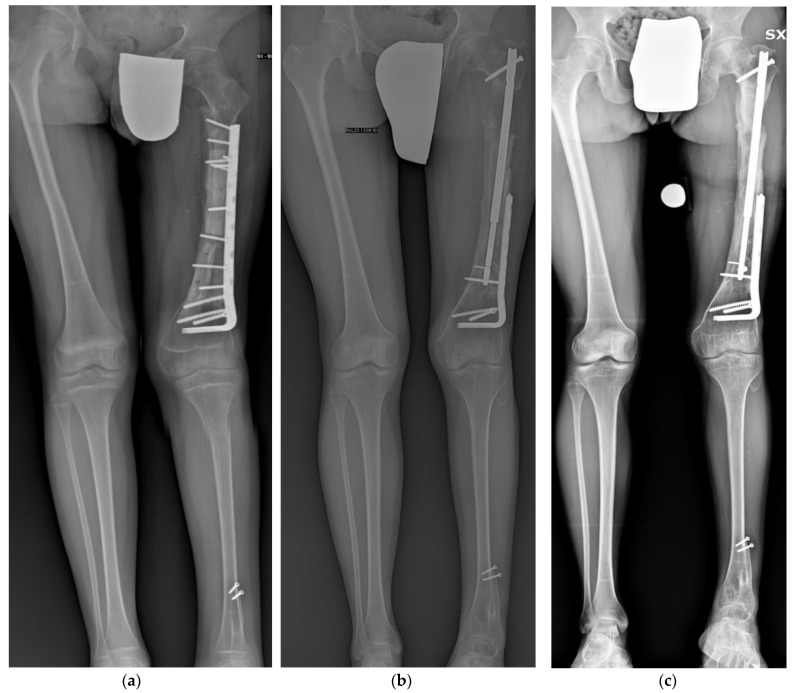
(**a**) Illustrative case of a 12-year-old male patient treated for distal femur osteosarcoma with femoral diaphysis resection and massive intercalary bone graft and vascularized fibula. The achieved LLD was 7 cm at the end of bone growth. (**b**) Antero-posterior panoramic lower limb radiography at the end of the lengthening. (**c**) Antero-posterior panoramic lower limb radiography showing complete healing 3 months after the lengthening. Patient presented a residual LLD of 2 cm and valgus.

The average patient age at the time of surgery was 19 (range 11–32). There were 9 men and 9 women. The average follow-up time was 29 months (range 12–59). PRECICE 2 nail treatment was performed on 15 femurs (7 retrograde and 8 anterograde) and 3 tibias. The average preoperative LLD was 50 ± 20 mm. The average achieved length was 38 ± 17 mm, while the average DI was 12 ± 4 days/cm. Lengthening details are shown in Table 1. All patients achieved regenerate consolidation at the end of the follow-up. Out of 18 patients, 16 (89%) reached the targeted length (Figure 1). In two patients, the prolonged length discrepancy induced a progressive equine rigidity; thus, the discrepancy was not completely corrected. Ten patients presented residual limb length discrepancy of 7.2 mm on average (range 5–20 mm).

The average consolidation time was 141 days (range 50–360) with a mean CI of 31 ± 12 days/cm. The ASAMI bone score showed 14 (82%) excellent results, 1 (6%) good result, and 2 (12%) poor results. The ASAMI functional score showed 13 (76%) excellent results, 2 (12%) good results, and 2 (12%) poor results (Table 2).

### 3.2. Role of Chemotherapy

Patients treated with chemotherapy for bone cancer did not show an increase in distraction for consolidation time. Moreover, they presented the same risk of complications. No statistical difference was observed in the CI for the tibia or femur. Gender had no influence on consolidation time.

### 3.3. Difficulties

A total of seven (18%) problems, one obstacle (2%), and three complications (8%) were encountered in our case series.

### 3.4. Problems

According to the Paley classification, we found three problems. Three patients (8%) presented with delayed bone healing (one tibia, two femurs) but without requiring further surgical treatment to foster bone healing. No delayed union was reported at the end of the follow-up. No cases of deep or superficial infection were observed. Four patients (10%) suffered knee stiffness and limping, making it necessary to slow down the distraction rate and intense physiotherapy.

### 3.5. Obstacles

We encountered one obstacle. One patient presented a fracture distally to the distal screw of the femoral nail that healed conservatively.

### 3.6. Complications

Three complications were observed. One patient presented hip endoprosthesis subluxation during lengthening; however, lengthening was performed as scheduled until the end of lengthening.

**Figure 2:**
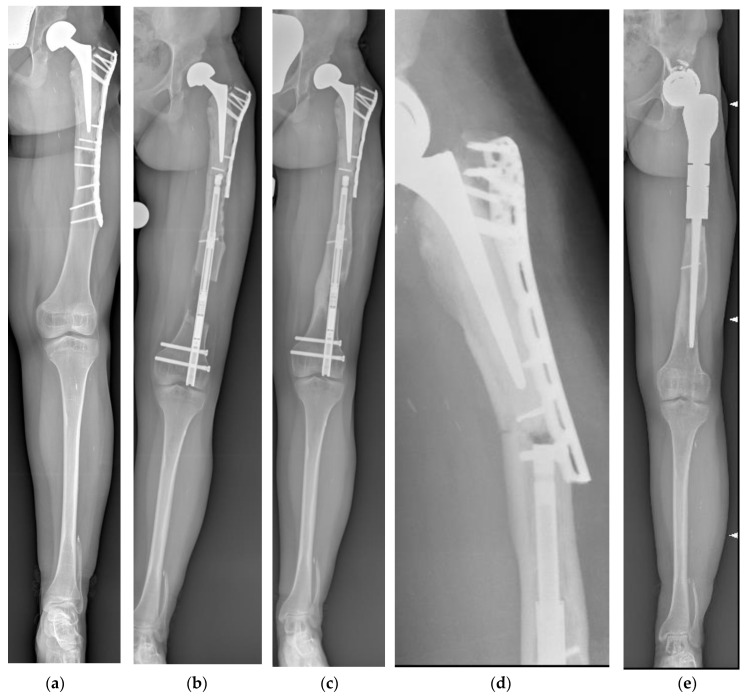
(**a**) Antero-posterior panoramic lower limb radiography of a 15-year-old female patient treated for left proximal femur osteosarcoma when she was 4 years old. She underwent proximal femur composite endoprosthesis. The achieved LLD was 5 cm. The available space for the nail was only 19 cm, while the shortest one was 215 mm. We had to cut 1 cm off of the telescopic part of the nail to minimize the protrusion as much as possible. The patient did not mention pain during knee extension or flexion. (**b**) Antero-posterior panoramic lower limb radiography showing hip endoprosthesis subluxation during lengthening. Lengthening was performed as scheduled until the end of lengthening. (**c**) During the consolidation phase, the running back phenomenon was observed, and the nail was shortened by 1 cm. Complete bone healing was observed 6 months after the end of lengthening. (**d**) Three years after the end of lengthening, the patient experienced a fracture proximally to the femur and reconstruction with a composite endoprosthesis, and (**e**) a proximal femur megaprosthesis was implanted.

One femur fractured proximally to the nail 36 months after the end of lengthening. The patient was previously treated with a proximal femur resection and reconstruction with a composite endoprosthesis. After the fracture, the previous reconstruction was removed, and a proximal femur megaprosthesis was implanted. A case of 1 cm nail shortening during the consolidation phase was observed (Figure 2).

No nail integrity breakage occurred during our study period.

## 4. Discussion

Limb length discrepancy is a common outcome in patients treated for benign or malignant bone tumors during childhood. Patients treated for bone cancer underwent extensive bone resection and reconstruction with a massive bone graft or megaprosthesis and chemotherapy. Both elements make these patients more prone to complications. There are limited reports on the use of the PRECICE 2 system in patients with a history of bone tumors [18,19,20,21]. To the best of our knowledge, this is the largest series reporting the use of an intramedullary magnetic lengthening nail in cancer patients. Based on the assumption that the aim of intramedullary nail lengthening is to achieve leg lengthening with faster rehabilitation and a lower risk of complications [12,15], we consider whether functionality and outcomes support these concepts.

### 4.1. Limitations

We recognize several limitations in the current study. First, this is a retrospective analysis of a consecutive cohort of patients. Moreover, there is no control group, and the number of patients is limited. We acknowledge that patients were not randomized and that only descriptive statistical analyses were performed. The results may be biased by the fact that patients were selected for this procedure. In consideration of these limitations, conclusions from the current study can be seen only as a general trend.

### 4.2. Lengthening

We found that the targeted length was obtained in 94% of cases. These data are consistent with the data reported in the literature [22,23,24,25,26] for this device. In particular, Schiedel et al. [24] reported an accuracy ratio of 97% in a series of 26 implanted nails. Nasto et al. [23] described an accuracy ratio of 91.1% in a series of 26 patients. Comparable results were reported by Wagner et al. [27], who described an accuracy ratio of 97.7% in a series of 30 patients. Moreover, patients treated with an intramedullary magnetic nail have less pain after the initial postoperative period and require less intensive physical therapy support, thus permitting toe-touch weight bearing only a few days after surgery. Magnetic lengthening combines the complete control of daily lengthening with greater ease of use.

We can report that all patients achieved regenerate consolidation at the end of the follow-up without needing bone grafting, bone marrow, or any other bone inducer to achieve full bone callus formation. These data are better than those of a previous study that used the first-generation PRECICE nail. Paley [17] reported an incidence of delayed union in 3 out of 65 (4.6%) patients treated with a PRECICE nail, and Kirane et al. [22] reported 8% delayed bone healing.

Even though tibial lengthening presented a higher risk for nonunion or has been reported as requiring a longer time to consolidate [28,29], we did not find a significant correlation between tibial lengthening and delayed union. These data may be related to the low number of tibias in our cohort.

The ASAMI bone and functional scores showed excellent results in 82% and 76% of the cases, respectively. Three patients suffered knee stiffness and limping at the end of lengthening. In all cases, the distraction rate was reduced, and an intensive physiotherapy program was started. All patients regained their preoperative joint range of motion and showed a normal gait pattern a few months after the end of lengthening. Joint stiffness is a common complication in limb lengthening. Previous papers reported comparable results in postoperative function using the intramedullary nail lengthening devices Fitbone*^®^* [30,31] and PRECICE nail [22].

A potential drawback of intramedullary lengthening is the reaming of the intramedullary canal. This may disturb bone formation in a distraction gap because of the interruption of the endosteal blood supply. The importance of endosteal blood supply was underlined by Ilizarov, who deemed external fixators better for bone healing [32,33]. Donnan [34] described an average CI of 43.6 days/cm in a series of 41 children treated (57 procedures) with an external fixator. Intramedullary lengthening presented lower CI. Krieg et al. [31] described a CI of 26 days/cm in a series of eight patients treated with Fitbone TAA. The PRECICE 2 nail presented a lower CI compared to most devices. Nasto et al. [23] reported a CI of 25.1 days/cm, and we found a mean CI of 31 days/cm. We may surmise that the damage to the intramedullary canal does not affect the bone consolidation potential. On the contrary, the ability to carefully control daily lengthening is an important factor in obtaining a good regenerate. Kenaway et al. [35] stressed that a distraction rate greater than 1.5 mm/day is a predisposing factor to poor regenerate.

### 4.3. Role of Chemotherapy

We found that patients treated with chemotherapy for malignant bone tumors did not show increased CI. These data, to our knowledge, have not been reported in the literature thus far. On the other hand, having undergone chemotherapy is not a contraindication for intramedullary nail lengthening.

Patients treated for bone tumors are more prone to developing a deep infection secondary to the immunosuppressive effect of neoadjuvant and adjuvant chemotherapy; long operating times; and reconstructions like megaprostheses, massive bone grafts, or composite prostheses [36,37,38]. Pin tract infection is a very common complication associated with external fixation. Riganti et al. [19] reported infection in 78% of 32 patients with LLD treated with external fixation. Similarly, Pesenti et al. [39] described pin tract infection in 51.6% of cases, while Eidelman et al. [40] saw it in 45%. Even if superficial, pin tract infection may require antibiotics and cause a deeper infection that requires surgical treatment. Because it does not use external devices that penetrate the skin, an intramedullary nail helps minimize the risk of infection.

### 4.4. Difficulties

The absence of a standardized complication classification limits the ability to compare the rate of complications across studies (Table 3). The following are the difficulties we found according to the Paley classification [41].

Joint stiffness is a common complication in limb lengthening, and we observed four cases of joint stiffness that resolved after distraction rate reduction and the start of intense physiotherapy. These data are consistent with other series [23]. However, intramedullary lengthening is better tolerated by patients compared to lengthening with an Ilizarov frame or another external fixation.

Complications may be categorized as follows:

- Failure of the distraction mechanism;
- Failure of the nail’s integrity;
- Complication related to the previous treatment for a bone tumor.

In our cohort, we did not observe any cases of lengthening mechanism failure. These data are consistent with the literature. Nasto et al. [23] reported no lengthening mechanism failure in a series of 26 PRECICE nails, and similar findings were reported by Kirane et al. [22] and Lee et al. [43].

In patients with a history of bone resection and reconstruction for bone tumors, the previous treatment impacts the lengthening strategy as well as the site of nail insertion, the nail size (diameter and length), the osteotomy seat, and whether the previous synthesis may need any changes. One patient experienced the running back (RB) phenomenon, hip subluxation, and a fracture proximally to the nail. RB consists of an acute shortening of the device and the regenerate. It may or may not be associated with the breakage of the rotation coupling of the nail, resulting in rotational instability. This is a well-known complication in PRECICE 2 nails and also affects other mechanical devices [23,31,43,44]. In our RB case, we observed only shortening without any rotational instability. The patient had previously been treated with a proximal femur composite endoprosthesis when she was 6 years old. Lengthening was performed 8 years later, and subluxation was expected from the beginning of lengthening. Acetabulum resurfacing was planned and performed 1 year after the end of lengthening. These data are consistent with previous studies. Wagner et al. [27] described 1 out of 32 patients, and Szymczuk et al. [45] described 2 cases in a series of 30 nails; other studies include [17,23,46].

The same patient experienced a fracture between the nail and the previous reconstruction. To perform intramedullary lengthening, we decided to remove the screws and shorten the plate in hopes of avoiding any mechanical interference during nail insertion. A retrograde femoral nail was used, the osteotomy was performed distally to the femur, and the nail was proximally fixed in the allograft. Plate shortening had increased the stress in the transition area between the nail and the previous reconstruction, leading to the fracture. Shortening hardware provides limited benefits. In patients with a comparable reconstruction, lengthening was successfully performed without plate shortening. In other words, the surgeon must preserve the first reconstruction as much as possible and avoid a stress riser by leaving unprotected bone. To the best of our knowledge, this has never been addressed in the literature thus far.

## 5. Conclusions

Our data show that the PRECICE 2 nail system (1) allows effective and accurate lengthening preserving the range of motion in patients treated for bone tumors. (2) Even if intramedullary nail lengthening is not completely without complications, the risk of complications is lower than that with other devices. The opportunity to start intense physiotherapy earlier should prevent permanent joint stiffness. On the other hand, the overall risk of complications using the PRECICE 2 nail appears to be lower than that with other devices, while the likelihood of a good outcome is higher. (3) Having undergone chemotherapy for a bone tumor does not increase consolidation time or distraction time. (4) The initial synthesis must be preserved as much as possible to avoid a stress riser by leaving unprotected bone.

## Figures and Tables

**Table 1:** Lengthening details.

Site	Age (y)/Sex	LLD Etiology	LLD (mm)	Gained Length (mm)	Residual LLD (mm)	CHT
Tibia	11/F	Proximal tibia composite prosthesis for OS	30	40	10	Yes
Femur (A)	13/M	Femoral intercalary reconstruction for OS	52	49	15	Yes
Femur (R)	15/F	Proximal femur composite prosthesis for OS	50	40	15	Yes
Femur (A)	15/F	Ollier disease	45	45	0	No
Femur (R)	17/F	Polyostotic fibrous dysplasia	80	50	10	No
Femur (A)	17/M	Multiple exostoses	30	30	0	No
Femur (A)	18/M	Femoral intercalary reconstruction for OS	70	60	28	Yes
Femur (A)	18/F	Proximal femur curettage and bone grafting for UBC	32	31	5	No
Femur (A)	18/F	Partial distal femur resection for Parosteal OS	30	45	0	No
Femur (R)	18/M	Pelvic reconstruction	40	40	10	Yes
Femur (R) (*)	18/M	Pelvic reconstruction	120	50 + 50	20 (**)	Yes
Tibia	19/M	Multiple exostoses	50	50	0	No
Femur (A)	19/F	Proximal tibia composite prosthesis for OS	40	30	10	Yes
Femur (R)	20/M	Femoral intercalary reconstruction for ES	70	70	12	Yes
Femur (R)	24/F	Proximal femur curettage and bone grafting for ABC	35	25	0	No
Tibia	25/M	Proximal tibia composite prosthesis for ES	100	80	30	Yes
Femur (R)	28/M	Rotationplasty	60	35	0	Yes
Femur (A)	32/M	Distal femur resection	45	50	0	Yes

LLD; lower limb discrepancy; (A): anterograde nail; (R): retrograde nail; OS: osteosarcoma; ES: Ewing sarcoma; UBC: unicameral bone cyst; ABC: aneurysmal bone cyst; CHT: chemotherapy before lengthening; (*) patient 11 underwent lengthening twice; (**) residual LLD after the second lengthening.

**Table 2:** Evaluation of the clinical outcome according to the ASAMI classification.

ASAMI Bone Score	Number of Patients	%	ASAMI Functional Score	Number of Patients	%
Excellent	14	82%	Excellent	13	76%
Good	1	6%	Good	2	12%
Fair	0	0%	Fair	0	0%
Poor	2	12%	Poor	2	12%

**Table 3:** Complication rate in major series reported in the literature according to different methods of treatment. DI: Distraction Index; CI: Consolidation Index; NR: Not reported.

	Device	Number of Limbs	DI (Days/cm)	CI (Days/cm)	Delayed Consolidation No. (%)	Stress Fracture	Implant-Related Complication (%)
Dinçyürek et al., 2012 [30]	FITBONE	15	12	43.7	3 (20%)	0	13.3%
Krieg et al., 2011 [31]	FITBONE	32	10.6	41.5	2 (6.25%)	0	12.5%
Kirane et al., 2014 [22]	PRECICE	24	NR	NR	2 (8.3%)	0	4%
Wagner et al., 2017 [27]	PRECICE	30	22.4	36.4	4 (13.3%)	0	0
Accadbled et al., 2019 [42]	FITBONE	8	NR	48.4	NR	0	18%
Nasto et al., 2020 [23]	PRECICE 2	26	11.9	25.1	2 (7.69%)	1	3%
Present Study	PRECICE 2	18	12	31	3 (17.64%)	1	5%

## Data Availability

The data presented in this study are available on request from the corresponding author. The data are not publicly available due to privacy restrictions.
